# Archaea appropriate weaponry to breach the bacterial cell wall

**DOI:** 10.1371/journal.pbio.3003323

**Published:** 2025-08-15

**Authors:** Anna C. Papageorgiou, Panagiotis S. Adam

**Affiliations:** Institute of General Microbiology, Kiel University, Kiel, Germany

## Abstract

Archaea will sometimes turn antagonistic against Bacteria using hitherto unknown mechanisms. This Primer explores a new study in PLOS Biology that pioneers a homology-based approach to discover bacterium-killing peptidoglycan hydrolases in archaea.

## Teaser

Archaea will sometimes turn antagonistic against bacteria using hitherto unknown mechanisms. A new study in *PLOS Biology* pioneers a homology-based approach to discover bacteria-killing peptidoglycan hydrolases in archaea.

After their split at the Last Universal Common Ancestor, bacteria and archaea followed different evolutionary trajectories for multiple systems. One of them was their respective cell envelopes. Archaea can produce a variety of structures, such as S-layers, pseudomurein, and methanochondroitin. Bacteria almost universally have a peptidoglycan cell wall. This nigh-ubiquity makes peptidoglycan and its biosynthesis prime targets for antibiotics, like β-lactams and antimicrobial peptides [1] that could circumvent antibiotic resistance. Through an assortment of enzymes called peptidoglycan hydrolases (PGHs), bacteria remodel peptidoglycan throughout their life cycle, for example, to release and reuse soluble peptidoglycan fragments during growth or for the separation of daughter cells during cell division. Certain PGHs can be secreted as lytic agents in predation or against specific members within a population [2]. A new study by Strock and colleagues [3] set out to discover unknown PGHs in Archaea that could mediate antagonistic interactions with Bacteria. To ensure the target specificity toward peptidoglycan, they introduced a homology-based approach where the search was constrained to proteins containing at least one catalytic (amidase, peptidase, or glycosidase) domain and one peptidoglycan-binding domain.

The study makes headway in addressing the question of how bacteria and archaea interact; particularly the extent and underlying mechanisms of cross-domain conflict. Interactions between members of the two domains usually run the gamut from commensalism to competition for resources [4]. Nanosized bacteria with reduced genomes from the Patescibacteria phylum have even been found to leapfrog the domain divide, acting as putative epiparasites [5] of archaea. However, antagonistic interactions are almost undocumented. Archaea do produce antimicrobial peptides and proteins that usually target other archaea. Among them are archaeocins that are also encoded by some bacteria [6]. Another peculiar case is a CAMP-like peptide that is effective against bacteria, encoded by the hybrid plasmid-virus element pSSVx of the archaeaon *Sulfolobus islandicus* [7].

When it comes to attacking the peptidoglycan, a Glycoside Hydrolase Family 25 muramidase PGH has been independently acquired by a few eukaryotes and the archaeon *Aciduliprofundum boonei* through horizontal gene transfers from bacteria. This archaeal homolog was shown to have antibacterial activity [8]. PGHs in Archaea could be directed either against living bacteria for predation or necrotrophy, or used for scavenging environmental peptidoglycan. Strock and colleagues searched for PGHs in a representative set of prokaryotic genomes from the Genome Taxonomy Database. The constraint of both a catalytic and peptidoglycan-binding domain in the same protein made the search specific to PGHs instead of lytic enzymes with other potential targets, but also probably limited the bacterial targets mainly to Gram-positive bacteria whose PGHs have such modular architectures. The structure of the peptidoglycan layer differs between Gram-positive and Gram-negative bacteria. Or more properly, “monoderms” with one membrane and “diderms” with two membranes, although there are exceptions. Even lineages in the same category can exhibit differences in peptidoglycan composition, structure, and interactions with the membrane(s) [9,10]. Almost 5% of archaea were found to possess at least one such modular PGH.

To limit the space for further validation, the authors zeroed in on archaeal homologs of zoocin A, which is well-documented as a bacteriolytic. The M23 peptidase domain of zoocin was found in 10 archaeal genomes, half of which belong to the class Halobacteria. The halobacterial homologs consist of an N-terminal M23 peptidase domain and two C-terminal peptidoglycan-binding domains. The domain order is inverted from the closest bacterial relatives of the M23 peptidase, where it is linked to a LysM domain, indicating domain shuffling in PGH evolution. Each domain type (peptidase, peptidoglycan-binding) was acquired from different donor lineages in Bacteria through independent horizontal gene transfer events. The patchy taxonomic distribution probably reflects frequent gene gain and loss events, as PGH necessity waxes and wanes through competition-driven evolution.

The chimeric zoocin A PGH from *Halogranum salarium* B-1 carries a Tat-signal peptide, indicating that it is secreted. Strock and colleagues named it Woldo, after a historical Korean weapon. Based on sequence identity and predicted structural similarity for the peptidoglycan-binding domain that should confer target-specificity, they picked three cultured halophilic bacteria for testing the activity of Woldo. Due to growth condition overlaps, these bacteria could, in principle, encounter *H. salarium* in their habitat. *Haloferax volcanii* supernatant producing Woldo by heterologous expression had bactericidal activity against *Halalkalibacterium halodurans* among the three candidate targets. The bactericidal activity was also observed for *H. salarium* supernatant. However, proteomic analysis indicated that another PGH was produced in high abundance. The second PGH consists of a glucosaminidase domain fused to the two peptidoglycan-binding domains and also carries a Tat-signal peptide. It was named Danwoldo, after a weapon related to Woldo. Danwoldo can also kill *H. halodurans* when produced by *H. volcanii*.

Bioprospecting for peptides and enzymes, including antimicrobials, through phylogenetic methods and massive (meta)genomic data has become commonplace. Machine learning methods are now into the fray to mine *en masse* for small open reading frames encoding antimicrobial peptides [11]. Strock and colleagues add an ingenious spin with domain presence and homology constraints. Although they focused on the bactericidal activity of PGHs from a cultured archaeon targeting cultured bacteria, the success of their *in vitro* pilot study suggests that PGHs or other antibacterial enzymes could be found through metagenomic data. We suggest that the search for targets could further be constrained to bacterial metagenome-assembled genomes (MAGs) from the same metagenome as the archaeal PGHs, so one could mine all antagonistic interactions in that environment. The authors found that the biome correspondence between the archaea encoding PGHs and their predicted target bacteria is non-random.

Finally, the authors propose a 4-fold approach to explore archaeal-bacterial antagonistic interactions. First, their strategy could be adapted for other systems separated at the bacterial–archaeal divide. For example, searching for archaeal enzymes that interact with bacterial membrane phospholipids, or presumably outer membrane tethering systems for diderms [10]. Bacteria employ a wide array of other systems, from bacteriocins to contractile injection systems, including type VI secretion systems, to attack their kin, but there has been little study as to the extent that archaea have adopted these systems and whom they target. There might even exist novel systems syntenic to genes coding for proteins with antibacterial function. Finally, new antibacterial proteins and small molecules will be discovered by bottom-up experimental approaches such as co-cultivation. While there has been a recent push toward *de novo* protein design through machine learning [12], in our opinion, there exists promise in marshaling classic evolutionary and phylogenetic approaches, such as directed evolution [13] and ancestral sequence reconstruction and protein resurrection [14]. Regardless of the road taken in bioinformatics, experimental validation and determination of the underlying mechanisms through biochemical characterization and integrative structural biology will remain the final step, the golden standard, but also the tightest bottleneck in antibacterial discovery.

## Abbreviation

MAGs: metagenome-assembled genomes
PGHs: peptidoglycan hydrolases
